# HIV and an Ageing Population—What Are the Medical, Psychosocial, and Palliative Care Challenges in Healthcare Provisions

**DOI:** 10.3390/microorganisms11102426

**Published:** 2023-09-28

**Authors:** Mohamed H. Ahmed, Fatima Ahmed, Abu-Bakr Abu-Median, Maria Panourgia, Henry Owles, Bertha Ochieng, Hassan Ahamed, Jane Wale, Benjamin Dietsch, Dushyant Mital

**Affiliations:** 1 Department of Medicine and HIV Metabolic Clinic, Milton Keynes University Hospital NHS Foundation Trust, Eaglestone, Milton Keynes MK6 5LD, UK; 2 Department of Geriatric Medicine, Milton Keynes University Hospital NHS Foundation Trust, Eaglestone, Milton Keynes MK6 5LD, UK; 3 Tele-Geriatric Research Fellowship, Geriatric Division, Family Medicine Department, Michigan State University, East Lansing, MI 48824, USA; 4 Leicester School of Allied Health Sciences, Faculty of Health and Life Sciences, De Montfort University, Leicester LE1 9BH, UK; 5 Department of Palliative Medicine, Milton Keynes University Hospital NHS Foundation Trust, Eaglestone, Milton Keynes MK6 5LD, UK; 6 Department of HIV and Blood Borne Virus, Milton Keynes University Hospital NHS Foundation Trust, Eaglestone, Milton Keynes MK6 5LD, UK

**Keywords:** HIV, aging, psychosocial, palliative medicine

## Abstract

The continuing increase in patient numbers and improvement in healthcare provisions of HIV services in the UK, alongside the effectiveness of combined antiretroviral therapy (cART), has resulted in increasing numbers of the ageing population among people living with HIV (PLWH). It is expected that geriatricians will need to deal with many older people living with HIV (OPLWH) as life expectancy increases. Therefore, geriatric syndromes in OPLWH will be similar to the normal population, such as falls, cognitive decline, frailty, dementia, hypertension, diabetes and polypharmacy. The increase in the long-term use of cART, diabetes, dyslipidaemia and hypertension may lead to high prevalence of cardiovascular disease (CVD). The treatment of such conditions may lead to polypharmacy and may increase the risk of cART drug–drug interactions. In addition, the risk of developing infection and cancer is high. OPLWH may develop an early onset of low bone mineral density (BMD), osteoporosis and fractures. In this review, we have also provided potential psychosocial aspects of an ageing population with HIV, addressing issues such as depression, stigma, isolation and the need for comprehensive medical and psychosocial care through an interdisciplinary team in a hospital or community setting. OPLWH have a relatively high burden of physical, psychological, and spiritual needs and social difficulties, which require palliative care. The holistic type of palliative care that will improve physical, emotional and psychological wellbeing is discussed in this review.

## 1. Introduction

The gradual expansion of HIV services globally and the rapid therapeutic and management advances seen over the last four decades has meant that PLWH are living beyond the age of 50 years, provided that they receive quality-assured monitoring and surveillance of their HIV infection [1,2]. Different types of cART are widely used, with the desired aim of achieving the undetectable HIV-1 viral load seen in most cohorts of PLWH [3]. However, living longer can be associated with observed increases in different medical conditions, as well as cART being associated with different side effects [4,5], and this is reflected in PLWH in both resource-limited and rich countries. This may also be associated with risk of developing neurodegenerative disease, dementia, diabetes, obesity, chronic kidney disease, osteoporosis, frailty, and some cancers [1,6,7,8,9,10]. The risk of fracture also increases in association with osteoporosis and the increased risk of falls [11]. OPLWH are likely to be isolated, and this makes them more prone to mental-health-related illnesses such as anxiety, depression and addiction. This can further cause significant increases in social issues related to stigma, isolation, housing availability and ongoing social support in any one community [12,13].

These age-related issues are expected to increase in OPLWH in the near future. The success of the UNAIDS statement of achieving global 90-90-90 targets (90% aware of their status, 90% on HIV medication, and 90% have viral suppression) has further highlighted the needs of this particular cohort and indeed, a more ambitious 95-95-95 target is hoping to be achieved by 2030. The programme started in 2014 and just eight countries met the targets in 2020. In 2021, the 95-95-95 target was achieved in two countries [2,14,15]. It is estimated that one-fifth of PLWH globally are above the age of 50 years old (around 7.5 million) [2]. Currently, half of total PLWH in the USA are above the age of 50 years old, 15% of PLWH are over 50 years old in sub-Saharan countries and in the Netherlands the number of PLWH aged 50 years is expected to reach 70% by the year 2030 [5].

Despite the improvement in the medical care and treatment of HIV, significant numbers of PLWH die from HIV and HIV-related complications. In systematic reviews and meta-analyses, it was shown that the pooled proportion of patients readmitted to hospital after discharge was 18.8% (95% CI 15.3–22.3) and the percentage who died post-discharge was 14.1% (10.8–17.3). This analysis was conducted in 29 cohorts, including 92,781 PLWH. The study showed that inpatient treatment with cART during hospitalisation was protective against post-discharge mortality. Higher mortalities were seen in Africa (23.1% [16.5–29.7]) than in the USA (7.5% [4.4–10.6]). Importantly, mortality risk factors were associated with a long hospital stay, discharge against medical advice, low CD4 cell counts at admission and lack of follow-up care after discharge [16]. Palliative care is needed to provide symptomatic control such as pain management, anxiety, and nausea, which will further improve wellbeing. Palliative care training also needs to be offered to health professionals caring for OPLWH [17,18]. In the near future, geriatric and palliative care services will need to expand in order to meet the demand of the ageing population of people living with HIV. The objective of this review is to highlight the most important medical, psychosocial and palliative care challenges. We have also attempted to suggest possible strategies for such challenges.

## 2. Methods

This narrative review article was conducted using the following databases: Medline, Scopus, PubMed and Google Scholar. These databases were searched using the keywords HIV, ageing, elderly population, social, palliative and medical condition in older HIV populations. The search was based on studies published in English from January 2000 to June 2023. We included only randomised controlled trials, clinical trials, systematic reviews and original articles in the study. We have excluded letters to the editor, commentaries, case reports, opinions, protocols, news, theses, notes, short surveys, conference abstracts and repeated studies.

### 2.1. Medical Challenges

HIV can be associated with different and serious medical conditions that can lead to fatal outcomes. The medical conditions will represent extra challenges for geriatricians providing care within the service, which is already overwhelmed with similar conditions in patients without HIV and these are due to the following [19,20,21,22,23,24,25,26,27]:

1. Lack of global plans to establish quality-assured geriatric HIV services.
2. Scarcity of clinical trials available in elderly populations living with HIV.
3. No clinical guidelines are available about the best strategies in the management of medical conditions in elderly populations living with HIV.
4. Issues in relation to geriatricians.
  - Global shortage of trainee and accredited geriatricians.
  - Extreme shortages of geriatricians trained in HIV care and a lack of specific provisions in infectious diseases and related training schemes to provide in-depth knowledge in this area.
  - No clear consensus about a defined age or range a patient will need to see geriatricians, as it can range broadly from the age of 50 or 65 years old.
  - The screening tools used in geriatric medicine are not yet validated in/for HIV populations.
5. Lack of needed guidance for intensive care physicians about who will and will not benefit from admission to intensive care units.
6. Lack of guidance for primary care physicians in the management of medical conditions in elderly populations with HIV.
7. Guidance and consensus are needed for community geriatric service.
8. The need for hospital and community pharmacists to be educated in dealing with drug interactions.
9. Lack of training in HIV care for occupational therapists, physiotherapists and other allied healthcare specialties, e.g., dietitians and podiatrists.
10. Provision of extra funding and capacity building for all the reasons noted above.

#### 2.1.1. Neurocognitive Disease and Dementia

Alzheimer’s disease (AD) can also be seen in PLWH at middle or older age [28]. The risk of dementia will increase significantly at the age of ≥65 years [29]. The estimated prevalence was suggested to be 2–5% [29,30,31]. HIV-associated dementia (HAD) can be seen in ageing populations living with HIV, whereas HIV-associated neurocognitive disorders were more prevalent in the pre-cART era [32]. However, HIV-associated neurocognitive disorder (HAND) is seen in mild and subtle forms and can be difficult to diagnose. As both conditions can present similarly, both conditions can be classified and managed on best clinical practice, as no definitive treatment is available [33]. In a systematic review and meta-analysis, the prevalence of HAND was estimated to be 50.41%. Factors that can lead to HAND were low level of education, older age, advanced stage of the illness, low CD4 counts of <500 cells/μL and depression [34,35]. Age duration of HIV infection and low CD4 counts appear to represent the main factors leading to HAND [29]. Other factors such as HIV co-infection and other sexually transmitted diseases such as syphilis, hepatitis C and cytomegalovirus can also increase HAND prevalence [36,37]. Other non-communicable diseases that increase risks of HAND development are diabetes, hypertension, obesity and insulin resistance [38,39]. Previous and prolonged depression and apathy can also be contributing factors. The cART, efavirenz was also reported to be associated with cognitive impairment, along with other cARTs such as atzanavir, nevirapine, abacavir and etravirine, whereas less neurotoxicity was noted with tenofovir, darunavir and emtricitabine [40].

Dementia in HIV populations tends to affect fronto-striato-thalamo-cortical circuits. Therefore, in the early stages of HIV infection, individuals may be asymptomatic or develop hyperreflexia. With the progression of HIV and dementia, individuals may develop hypertonia, tremors and clonuses. Features related to Parkinsonism may infrequently develop with slowness in processing information as an important and early diagnostic feature of HAND [41,42]. This can be associated with mental slowness and attention and memory deficits. It is noted that episodic memory impairment with psychomotor slowing can be seen as sensitive indicators in HAND [43].

Regular screening for HAND using validated questionnaires can help in establishing the diagnosis. For instance, The European AIDS society (EACS) recommends regular screening using three questions: (1) Do you have memory loss? (2) Do you feel slowness in planning and solving a problem? and (3) Do you have a problem in paying attention? One positive answer to one of the three questions warrants further investigations. EACS also recommends excluding cofounding factors such as depression, substance abuse or excessive drinking of alcohol [44].

The screening tools used in HAD are the HIV dementia scale (HDS) and international HIV dementia scale (IHDS). The IHDS is easy to utilise and is widely used in different countries, especially where resources are limited. No previous training is required, and it accurately assesses memory, motor speed and psychomotor functions [45]. In a systematic review and meta-analysis, the IHDS showed a sensitivity of 79.4% and a specificity of 65.4% in detecting HAD [46]. The mini-mental state examination (MMSE) and the Montreal cognitive assessment (MoCA) can be used in HAD, but MoCA has more sensitivity and specificity than MMSE [47,48]. The Italian Society for Infectious and Tropical Diseases recommended screening all individuals with HIV for dementia and also recommended using the MoCA [49]. The British HIV Association and the American Association of Infectious Diseases also recommend screening for dementia in the new patients and then once per year [50,51]. No treatment for dementia is yet available, but in HAD the option will be treatment of underlying causes, optimisation of CD4 count through using cART and acetylcholinesterase inhibitors such as memantine [52,53].

#### 2.1.2. Bone Disease and Osteoporosis

HIV is a condition of chronic inflammation that is associated with a risk of osteoporosis. OPLWH are also at risk of dementia, and this may increase the risk of neglect, falls and bone fractures. The osteoporosis seen in PLWH can potentially begin at the age of 40 or 50 years old [11]. Importantly, HIV per se is an independent risk factor for bone fractures [54]. Hepatitis C virus (HCV) coinfection is associated with a greater risk of osteoporosis and fractures than HIV per se [55]. Some cARTs may also be involved in osteoporosis, such as tenofovir disoproxil fumarate (TDF) [56,57]. Therefore, is not surprising that the risk of fracture increases with HIV. For instance, the fracture risk was found to be 35 to 68% in comparison with the non-HIV-infected population [58,59]. Importantly, the prevalence of vertebral fracture prevalence was found to be 11.1% in PLWH [60]. In clinical practice, the use of the fracture risk assessment tool (FRAX) and measurement of bone mineral density (BMD) via a DEXA scan are widely used in HIV clinics around the world. Bisphosphonates in association with vitamin D and calcium replacement remains the main treatment [61]. Fractures can also be due to increased falls in OPLWH. The cost of living and managing fractures and osteoporosis in older people with HIV can add more burden to the healthcare systems due to the disability and need for special care.

#### 2.1.3. Falls

HIV is also associated with an increase in the risk of falls. For instance, the prevalence of falls is estimated to be around 12% to 41% [62]. Different studies have shown different related risk factors. Several studies showed an association with decreased physical functions and cognitive impairment [63,64]. OPLWH taking multiple medications can contribute to polypharmacy, which is also regarded as a risk factor for falls. This, in turn, can lead to an increase in co-morbidities that are also potentially linked to peripheral neuropathy [63,64,65,66,67]. Importantly, older age, female and white race were also associated with an increase in falls. The behaviour of the individuals may increase the risk of falls [65,66,67], for instance, substance abuse, having pets and depression are linked with a high risk of falls [63,64,67]. It is plausible that OPLWH have low BMD, which also makes them more at risk of having falls associated with fracture. The high risk of falls and fracture may increase the risk of lowered mobility and disabilities in the future. Therefore, falls and osteoporosis may represent a challenge in the management of PLWH.

#### 2.1.4. Frailty

Frailty is defined as “an ageing-related syndrome of physiological decline, characterised by marked vulnerability leading to adverse health outcomes” [68]. The symptoms associated with frailty include weakness and fatigue, medical complexities and reduced tolerance to medical and surgical interventions [69]. Frailty is an important issue in geriatric medicine. It is an independent risk factor for the development of cognitive decline [70]. Therefore, the clinicians in all disciplines need to have an awareness of frailty and the associated risks for adverse health outcomes; early identification can improve care for this most vulnerable subset of patients. Frailty is attributed to the fact that ageing causes dysregulation in the normal physiological responses to stressors, resulting in a lack of adaptation and dysfunction of the normal homeostatic responses [71]. Among the risk factors are age, duration of HIV infection >20 years, duration of cART use and smoking [71].

Sarcopenia, which is defined as decreased muscle mass, strength and performance, also shares features of frailty [72]. Sarcopenia prevalence in OPLWH is 24.1% in comparison with 11.1% in individuals without HIV [73]. In a multicentre AIDS cohort study (MACS) bone strength sub-study, the odds of frailty were 4.5 higher in the presence of sarcopenia. It was suggested that there is an interaction between frailty, sarcopenia and functional decline and that sarcopenia can potentiate frailty [74].

Part of the recommendations of the EACS is to screen for frailty once per year for those above 50 years old [75]. There are different tools that can be used to screen for frailty. For instance, The Chelsea and Westminster HIV Clinic in London, UK, published their 10 years of experience in an ageing population with HIV. They used clinical frailty score (CFS) (made up of nine grades, 1 very fit and 9 terminally ill, which is easy to use and widely applied and, most importantly, is a reliable score) and the cut off for referral to a dedicated HIV geriatric service was 5 [76]. HIV is regarded as independent risk factor for frailty; thus, the association of frailty and HIV is associated with high mortality and morbidity [77,78].

The main aim of treatment is the management of disabilities and co-morbidities and all associated geriatric syndromes. For instance, nutrition, exercise and treatment of all reversible causes, including sarcopenia. Frailty is always managed by a multidisciplinary team including geriatricians, HIV physicians, physiotherapists, dietitians, pharmacists, psychologists, social care providers and an occupational therapist if possible [71].

#### 2.1.5. Polypharmacy and Therapeutic Challenges

OPLWH on cART are aware that this is lifelong therapy. cART is usually given as a combination of different medications and, therefore, it is important for healthcare providers to be familiar with the side effects due to prolonged use. In addition, attention should be given to drug interactions and the risk of polypharmacy [79]. In the UK, the Liverpool HIV drug interactions checker (https://www.hiv-druginteractions.org/checker, accessed on 11 June 2023) is widely used in HIV clinics to assess the safety of other medications beside cART [80]. For instance, simvastatin is not recommended with cART, whereas statins that are not metabolised through the liver (e.g., rosuvastatin) may represent an alternative choice. Examples of the side effects of cART include older protease inhibitors that may cause hypertriglyceridemia, whereas the use of TDF is associated with risk of developing osteopenia and osteoporosis.

Overall, there is an inadequate representation of the older population living without HIV in related clinical trials. This represents a challenge for dedicated healthcare providers, as the pharmacodynamics and pharmacokinetics of medication may change in frail older populations and may cause unnecessary side effects. On some occasions, useful medications may not be given due to unpredictable side effects [81]. The same situations can also be applied to OPLWH; thus, it is imperative that careful revision of all medication should be carried out annually, especially for those >50 years old.

#### 2.1.6. Infections

HIV in an ageing population can have a serious impact on susceptibility to viral, bacterial and fungal infections. Ageing without HIV is a known risk factor for low immunity, and HIV per se is well known as a disease of the suppression of immunity. Therefore, in the ageing population living with HIV, infection is one of the serious causes of increases in mortality and morbidity. OPLWH may catch different infections such as tuberculosis, hepatitis, sexually transmitted diseases or other viral infections. In the UK, both The British HIV Association (BHIVA) and National Institute for Health and Care Excellence (NICE) advocate screening for latent TB infection (LTBI) and treating it, especially in high-risk individuals [82,83,84,85]. Hepatitis, in general, is one of the major causes of mortality and morbidity in HIV (risk of liver cirrhosis and liver cancer). Hepatitis A, B or C can cause severe and long-lasting liver disease in PLWH, and vaccination is recommended (no sufficient evidence of benefit) [86,87,88,89,90,91,92]. Cytomegalovirus (CMV) has been suggested as a co-factor for the rapid progression of HIV-1 disease, as well as inflammatory and immune response activation. End-organ CMV disease develops in high-risk HIV patients who present with common symptoms such as retinitis but can also involve multiple organs [93,94,95,96].

During the COVID-19 pandemic, older age accompanied by chronic comorbidities was associated with severe outcomes in patients with HIV. Other factors included an absence of ART, low CD4 count and unsuppressed HIV load. There was no increased risk of COVID-19 critical care (ICU and intubation) in patients older than 50 years compared with younger age groups. The risk of death in OPLWH due to COVID-19 potentially increases with co-infection with tuberculosis [97,98,99,100,101]. Other infections reported, such as sexually transmitted diseases, varicella zoster virus, pneumonia, urinary tract infections, fungal infections and parasitic infections [102,103,104,105,106,107,108,109,110,111,112,113,114,115,116,117,118,119,120,121,122,123,124]. Further studies are needed to assess the prevalence of these infections in OPLWH.

#### 2.1.7. Hypertension, Diabetes, Dyslipidaemia and Cardiovascular Disease

The risk of developing diabetes, hypertension, dyslipidaemia and cardiovascular disease increases with the duration of HIV and cART administration [1,6,8,9]. Unfortunately, the treatment of these conditions can lead to polypharmacy and significantly increase the risk of falls and fracture. Prolonged or regular hypoglycaemia may also enhance the risk of cognitive decline and increase the risk of cardiovascular disease. Diabetes per se can be associated with significant complications such as neuropathy, retinopathy, nephropathy and an elevated risk of developing cardiovascular disease [1,6,8,9]. Therefore, an HIV metabolic clinic is an integral part of HIV services. Our HIV metabolic clinic in Milton Keynes University Hospital showed promising outcomes in optimising parameters for the management of diabetes, dyslipidaemia and obesity [7]. Choosing the best cART in OPLWH is another medical challenge. The main aim will be to reduce side effects and have an adequate suppression of the viral load. Therefore, most global HIV-1 treatment guidelines suggest giving integrase strand transfer inhibitors (INSTIs) (such as bictegravir or dolutegravir) as a first-line treatment due to the reduced renal, bone and cardiac toxicities; this reduced toxicity is particularly important in an ageing patient. The nucleoside/nucleotide reverse transcriptase inhibitors (NRTIs) and non-nucleoside reverse transcriptase inhibitors (NNRTIs) also have a greater number of metabolic side effects, e.g., more effect on lipids and glucose compared with INSTIs aids this decision-making process (usually in the form of an MDT), with the latter also having a lower pill burden and fewer drug–drug interactions with other medicines, thus being particularly invaluable for polypharmacy issues [9].

### 2.2. Psychosocial Challenges

HIV is a chronic condition, and OPLWH are very likely to suffer from different psychosocial problems. In Table 1, we provide a summary and explanation for these psychosocial challenges. The main aim is to highlight as much as we can from these challenges. The detailed narrative of management will be difficult to accommodate in this review, as social circumstances, culture and customs in different parts of the world are not the same. Therefore, it is important that relevant stakeholders obtain relevant population- and cohort-based knowledge and consider implementation of potential proactive changes according to the needs of their own communities.

### 2.3. Palliative and End of Life Challenges

Palliative care for PLWH is vital and necessary, especially in the view of the increase in an ageing population with HIV. According to the last report of UNAIDS 2021, the mortality due to HIV has decreased to around 650,000, in comparison with two million people in 2004 [140]. Nevertheless, in observational analysis in the UK, it was shown that mortality rates are high in PLWH in comparison with the general population, particularly compared with non-AIDS infections (10.8, 9.8–12.0) and liver disease (3.7, 3.3–4.2). Late diagnosis of HIV was a strong predictor of death (hazard ratio (HR) 3.50, 95% CI 3.13–3.92), whereas early diagnosis was associated with a lower risk of death and most deaths as the result of an AIDS-defining illness. Importantly, all-cause mortality was highest in the year after diagnosis (SMR 24.3, 95% CI 23.4–25.2) [141]. Furthermore, data from meta-analysis showed that pooled non-AIDS causes of death prevalence estimates in high-income countries were 53.0%, in developing countries were 34.0%, and in sub-Saharan countries were 18.5%. Globally, cardiovascular disease, non-AIDS malignancies and liver disease are the main causes of disease in the HIV population [142]. The D:A:D study follow-up showed that liver disease and cardiovascular diseases are modifiable and, over time, are decreasing in prevalence, whereas no improvement was observed with non-AIDS cancer [143].

The challenge of cardiovascular disease in PLWH may go unnoticed in many centres unless specialists in the management of cardiovascular risk are deployed in management in dedicated HIV clinics. For instance, the HIV metabolic clinic in our centre in Milton Keynes University Hospital has proven to be successful [7]. This is crucial, as data from an observational study for a 10-year follow-up showed the prevalence of dyslipidaemia (29–52%, *p* < 0.001) and obesity (1.1–3.5%, *p* < 0.001) and a high prevalence of cardiovascular events and obesity with CD4 counts of <200 cells/μL [144]. The perception of care given to an ageing population living with HIV needs to be changed. For instance, data from the Dutch cohort modelling up to the year 2030 showed that the median age will increase to 56 years, those aged more than 50 years will increase to 73%, 84% will have at least one non-communicable disease (NCD) and these changes will be driven by the increase in cardiovascular disease prevalence [5].

Due to high mortality associated with HIV and the increase in co-morbidities, the palliative care in the ageing population will represent a cornerstone in the management of the elderly population living with HIV. The World Health Organisation (WHO) defines palliative care as “an approach that improves the quality of life of patients and that of their families who are facing challenges associated with life-threatening illness, whether physical, psychological, social or spiritual” [145]. Despite the fact that palliative care is regarded as a global human right, it not widely available around the globe (low availability of palliative care in low-resource countries) [146]. It is worth mentioning that the WHO recommended that HIV palliative care is an “essential component of a comprehensive package of care for people living with HIV/AIDS because of the variety of symptoms they can experience” [147].

**Figure 1:**
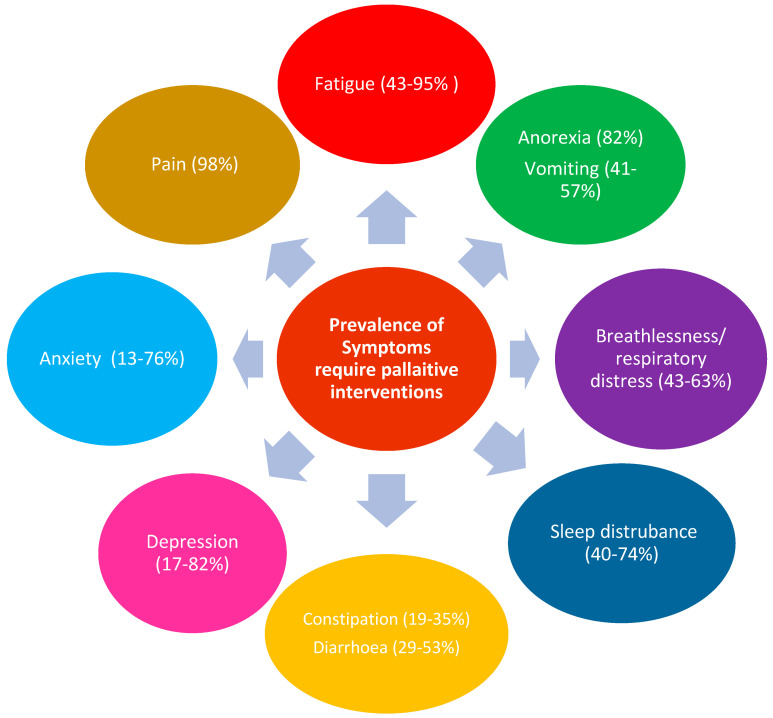
Prevalence of symptoms among PLWH that require palliative care interventions. References for sources of this figure are [17,18,126,136,137,138,139,148,160].

The benefit of palliative care in HIV and other conditions was shown in different studies, systematic reviews and meta-analyses. In Table 2, we present a summary of the randomised clinical trials, systematic reviews and meta-analyses and population studies about the benefits of palliative care in PLWH, whereas Figure 1 represents the prevalence of symptoms that require palliative care interventions. The incorporation of palliative care is urgently needed, as it represents an integral part of the HIV service for ageing populations. The complexity of the symptoms and their wide prevalence clearly represent a challenge for HIV physicians or geriatricians who are not trained in such a field. There is also a worldwide shortage in palliative care specialists, which in turn will represent a challenge in the near future. Unless proactive steps are taken to recruit and fund the training of doctors in this speciality, this could potentially lead to serious shortfalls in healthcare provision to this specialised population group. There are many challenges in providing palliative care for PLWH, as shown in Table 3. Based on these challenges, it is recommended that a person-centred model may represent the best option for delivering palliative care for PLWH.

**Table 2:** Summary of the main population studies about palliative care and HIV.

Theme and Article Type	Main Outcome	References
Palliative care and PLWH (systematic review)	Home palliative care and inpatient hospice care improved pain and symptom control, anxiety, insight and spiritual wellbeing.	[148]
Outpatient early palliative care (retrospective cohort study at an urban, academically affiliated HIV primary care clinic)	Common reasons for referral were chronic pain management and medication/appointment adherence. Retention in care improved significantly after the palliative intervention.	[149]
Need for palliative care in HIV with heart failure and cancer (nested case–control study, qualitative situational analysis study)	Urgent need to provide palliative care in cancer and heart failure patients with HIV.	[150,151]
Digital health and palliative care (systematic review)	Telemedicine was an effective health intervention in palliative care for PLWH in sub-Saharan Africa.	[152]
Benefits of palliative care in HIV (randomised clinical trial, systematic reviews)	Decreased cost, hospital admission, disease burden, exploring the individual’s needs, improve psychosocial distress, symptom control, holistic approach to patients and families and improve patient and caregiver satisfaction.	[153,154,155]
Early palliative care will improve wellbeing in PLWH and other co-morbidities (randomised clinical trial)	Variable improvement over time for three outcome variables, self-blame, symptom distress and HIV self-management, but no improvement in overall quality of life, social support or completion of advance directives.	[156]
HIV and depression, anxiety, stigma, mental health illness and post-traumatic stress disorder (review, systematic reviews)	High prevalence of psychiatric conditions and the need to screen for and manage them during the course of HIV.	[131,132,136,137,138,139]
Individuals’ issues that may represent a barrier to palliative care in people with HIV (systematic review)	Physical and psychological symptoms were highly prevalent at HIV diagnosis, wellbeing was impaired, suicidal thoughts were frequent and peace and calmness were reduced. Participants lacked emotional support and feared the reaction of their families. Practical problems included hunger, homelessness, reduced ability to work and the need for childcare.	[126]

**Table 3:** Challenges in palliative care for OPLWH.

Factor	Main Explanation	References
Patient factors	Unwillingness to accept discussion about end of life or use of medication to alleviate symptoms, such as opioids. The use of traditional healers in some Sub-Saharan countries and the South Asian subcontinent.	[17,18,126]
Hospital factors	Not all hospitals have the facility and staff members for palliative care. North American and European countries may be better equipped than middle- and low-resource setting countries.	[17,18,146]
Clinician factors	Not familiar with how to provide palliative care and manage symptoms. The majority of clinicians may have a disease-oriented approach rather than a palliative care approach.	[17,18,148]
Service factors	Availability of budget, resources and well-trained staff, justification of the need for the service (particularly in areas with a high prevalence of HIV).	[17,18,126,146,148]
herapeutic relationship factor	Noted in individuals with HIV and chronic diseases that they do not prefer to see new clinicians and prefer to be reviewed by clinicians they know.	[157,158]
Disease factors	Complex disease with different complex co-morbidities and clinical presentations that necessitate input from different medical specialities.	[1,6,7,9,11]
Spiritual and religious factors and those related to customs and traditions	Based on these factors, the most likely successful model will be the person-centred approach model.	[148,153,154,155]
Shortage of palliative specialists	There is a global shortage of palliative care medicine specialists.	[17,18]
Shortage of HIV specialists or geriatricians with an interest in palliative care	HIV specialists will develop holistic care relationships with related disciplines over years but are not currently trained in palliative care medicine. Geriatricians are also not trained in looking after ageing populations living with HIV. Future initiatives may consider how to incorporate palliative care services among clinicians that regularly care for PLWH.	[17,18]
Shortage of HIV nurses with speciality in palliative care	HIV nurse specialists will develop therapeutic relationships over the years and can be trained in palliative care medicine for ageing populations living with HIV, especially if incentives can be provided.	[17,18]
Paediatric palliative care	The evidence to support paediatric palliative care is still lacking; however, there is also an urgent need to look at this, especially in view of the 101,000 deaths among children as per the estimation of UNAIDS.	[159]

#### A Person-Centred Model of Palliative Care in HIV

PLWH develop holistic and therapeutic relationships with their healthcare providers over the many years of care and treatment provided. The person-centred model aims at addressing physical, psychological, social and spiritual concerns. Importantly, such an approach was found to decrease hospital admissions and cost and improve quality of life [160]. Clinical trials have shown that this model may provide more opportunities for assessment of the individual’s needs. For instance, in a randomised controlled trial (RCT) for pain management, treatment of constipation and discussion about spiritual worries was associated with increased emotional support and improvement in psychological wellbeing. This was attributed to the active ingredients of appropriate medication, effective health education and counselling and having time to talk in clinical encounters. The study endorsed the fact that health professionals providing care for HIV populations should make routine appointments more person-centred. This approach enabled staff to identify and manage multidimensional problems and provide tailored health education and counselling [161]. The person-centred approach has also been shown to improve mental health more than relieving pain. This is important in PLWH, as they tend to suffer from stigma and social isolation, especially among the older population. Palliative treatment can be delivered by a multidisciplinary team and palliative specialist input can be of a huge benefit, especially in complex cases [162]. However, person-centred assessment and care delivered by staff who have received additional training had positive effects on self-reported mental-health-related quality of life and psychosocial wellbeing [163].

The delivery of palliative care can be achieved within the setting of HIV outpatient clinics.

The emphasis is to deliver palliative care (supportive care, managing symptoms and psychosocial care) alongside HIV treatment, regardless of prognosis. For instance, in a prospective, longitudinal controlled design that compared patient outcomes at an outpatient facility that introduced palliative care training to clinicians and stocked essential palliative care drugs, pain was significantly decreased at the intervention site, with improvement in mental health, medical outcomes and physical score. Therefore, palliative care needs to be an integral part of the HIV service [164]. The model of a person-centred approach can be delivered through providing a two-week course about the general principles of palliative care for healthcare professionals in direct care for PLWH, as was shown in the TOPcare trial [165].

Perhaps it is possible to suggest that the person-centred approach may come with different benefits in PLWH: (i) it allows people to have choices and facilitates moving away from stigma and social isolation when they have the choice to open up discussion about end of life and place of death, (ii) the model also allows discussion of the needs of individuals in relation to their religion, spirituality, sexual orientation and traditional cultural practices, (iii) it allows addressing issues such as the management of pain, anxiety and sleep disturbances and (iv) it allows early integration of palliative care in an HIV service, as this can be initiated by health professionals working in HIV services and may allow for collaboration and excellent team work with palliative care specialists.

In the view of the increasing ageing population of PLWH, we need to shed light on the future of palliative care in HIV services. There is an urgent need for research and an audit to (i) assess feasible and cost-effective palliative care as integral part of HIV services, (ii) assess whether palliative care training can be offered to HIV physicians and geriatricians, (iii) undertake effective research and audits in low- and middle-income countries about the best ways to establish palliative care in such countries and (iv) form collaborations between palliative care specialists, HIV specialists, geriatricians and social scientists at national and international levels.

### 2.4. Comprehensive Geriatric Assessment

The comprehensive geriatric assessment (CGA) is part of the regular reviews conducted by geriatricians in the wards and during weekly multidisciplinary meetings. The main aim of the CGA is to provide multidisciplinary diagnostic and treatment processes that evaluate medical, psychosocial and functional deficits in order to provide coordinated interventions/plans that maximise overall health with ageing and the better use of the resources available [166]. Despite the fact that CGA data are limited in older PLWH, especially in outpatient settings, it is likely that CGA may improve outcomes in terms of functional outcome, pain control, mental wellbeing and nutrition [167].

**Figure 2:**
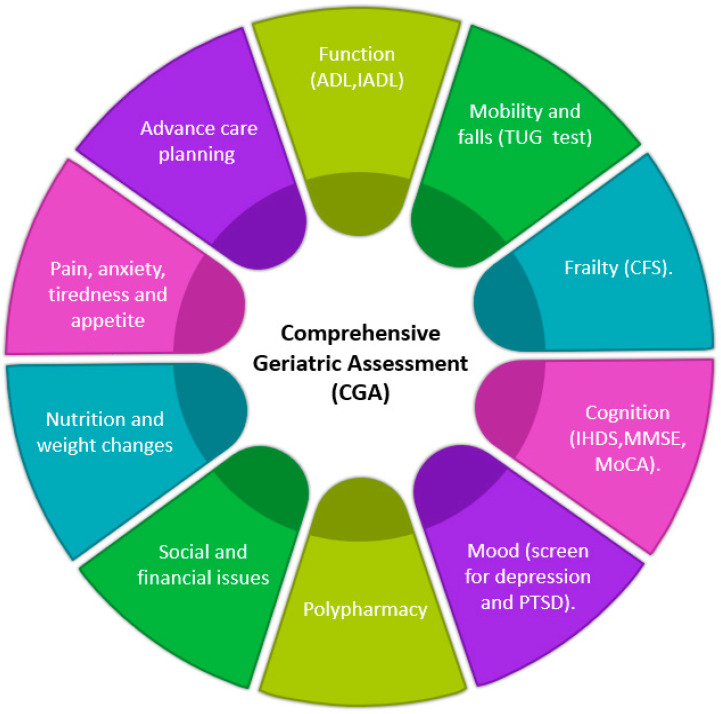
Components of the comprehensive geriatric assessment (CGA) in OPLWH. ADL (activities of daily living), IADL (instrumental activities of daily living), TUG (timed “Up & Go” test), CFS (clinical frailty score), IHDS (international HIV dementia score), MMSE (mini mental state examination), MoCA (Montreal cognitive assessment) and PTSD (post-traumatic stress disorder). References for sources of the this figure are [168,169,170,171,172,173].

In Figure 2, we showed the main components of the CGA in older PLWH. In the outpatient settings of an HIV service, the assessment can be achieved in two–three visits to meet all the designated targets. Function and mobility and falls can be assessed using screening tools such as activity of daily living (ADL), instrumental activity of daily living (IADL) and timed up-and-go (TUG) test [168,169,170,171,172,173].

Frailty can be assessed using CFS [77], whereas dementia screening can be achieved by using IHDS, MoCA and MMSE [46,48,49]. Importantly, HIV is a complex condition and use of cART can be associated with drug–drug interactions. Therefore, polypharmacy is best dealt with by a pharmacist specialising in HIV medicine [80,81,82]. The long interaction between older PLWH and an accredited HIV clinic may also provide an excellent opportunity to screen for pain, depression, post-traumatic disorders, burden symptoms of the disease (anxiety, lack of appetite, fatigue) and explore issues in relation to nutrition and weight changes.

The discussion about advance care planning is needed at some stage of the management of older PLWH. It is difficult to determine the timing of such a discussion, but potential candidates for such discussion are those aged more than 55 years or those with serious illness (clinicians’ discretion) [168]. The whole rationale is to allow for a documented discussion about an individual’s reflections and planning for future health plans when the individuals cannot make such decisions as he or she has encountered confusion or delirium associated with serious illness. This is crucial, as some relatives may not be aware of the individual’s HIV status and what their preferences are.

## 3. Conclusions

OPLWH are at risk of greater mortality and morbidity. A CGA is needed and can begin to be implemented in HIV outpatient clinics when the patient is at the age of 50 years or more. The CGA is essential, as its implementation will allow for early detection of all the medical and psychosocial issues discussed in this review and the establishment of a means of management. Importantly, CGA will also initiate essential discussion about advance care planning and this may in part decrease some of the challenges that face palliative care physicians in the management of a cohort in this population. Clinicians and advanced HIV nurses caring for OPLWH need to develop skills regarding when to refer individuals for elderly care or to palliative care physicians. We have developed an HIV metabolic clinic in Milton Keynes University Hospital, and such a clinic can also provide a point of referral for elderly care and palliative medicine, as it deals with cardiovascular risk. Assessment in a metabolic clinic may conclude that no further benefit can be achieved in terms of decreasing the metabolic and cardiovascular risk. There are holistic and therapeutic relationships that clinicians and healthcare professionals develop with patients over decades of management and care that may decrease stigma, anxiety and stress about living with HIV and related issues and current and future needs can be discussed in a confidential and empowering manner. Importantly, collaborations with other specialities and geriatricians and palliative care physicians are needed in order to manage complex conditions in OPLWH.

## Figures and Tables

**Table 1:** Summary of the main studies of psychosocial challenges.

Theme and Article Type	Main Outcome	References
Poverty and lack of basic minimum needs (cross-sectional study, systematic review)	High prevalence of poverty, especially in developing countries that mean majority are not able to meet need for housing, food and clothes.	[125,126]
Housing insecurity (systematic review)	Providing housing is a strong predictor of better medical care in HIV populations, as shown in randomised clinical trials (improved compliance with medication and decreased viral load and transmission).	[127]
Food insecurity (cross-sectional study)	68% have food insecurity, and this can be significantly associated with excess alcohol intake, immobility and depression.	[128]
Lack of employment (review article)	Poverty and lack of employment or sufficient savings may also contribute to the overall decrease in quality of life for OPLWH.	[129]
Immigration (population study)	Delay in diagnosis, poverty, accessibility to housing and food and HIV care.	[130]
Stigma (review articles)	The stigma for OPLWH can be related to gender, race, sexual orientation and socioeconomic status, and in older people with HIV this can be due to ageing. This may result in the following: Effect on mental and physical health (anxiety, depression, post-traumatic stress and isolation). Fear to share HIV status. Fear to seek help, treatment and socialisation with people.	[131,132]
Living alone and no social support (review articles and population study)	Isolation is one of the main problems for older people, not only for OPLWH.	[129,130,131,132]
Substance abuse (prospective observational cohort study)	High mortality among those with excess alcohol intake and long duration of smoking and depression.	[133]
New diagnosis of HIV in elderly populations and its related social issues (retrospective analysis, analysis of surveillance data)	New diagnosis can be associated with high mortality. There is a need for intensive emotional support and counselling as they need to adapt to new lifestyles, coping with new diagnosis of HIV, family relationship and being able to overcome medical complications of HIV, being diagnosed with sexually transmitted disease or hepatitis.	[134,135]
Mental health illness (depression, suicide and anxiety) and post-traumatic stress disorder (systematic reviews)	Different systematic reviews showed high prevalence of these conditions and the need to screen for and manage them during the course of HIV. Systematic reviews showed anxiety at 36–95% and depression at 18–47%.	[136,137,138,139]

## Data Availability

This review articles and information obtained from listed references.
